# Automatic classification of nerve discharge rhythms based on sparse auto-encoder and time series feature

**DOI:** 10.1186/s12859-022-04592-3

**Published:** 2022-02-15

**Authors:** Zhongting Jiang, Dong Wang, Yuehui Chen

**Affiliations:** 1 School of Information Science and Engineering, University of Jinan, Jinan, 250022 China; 2 Shandong Provincial Key Laboratory of Network Based Intelligent Computing, Jinan, 250022 China

**Keywords:** Nerve discharge, Classification, Neural network, Auto-encoder, Softmax, Feature learning

## Abstract

**Background:**

Nerve discharge is the carrier of information transmission, which can reveal the basic rules of various nerve activities. Recognition of the nerve discharge rhythm is the key to correctly understand the dynamic behavior of the nervous system. The previous methods for the nerve discharge recognition almost depended on the traditional statistical features, and the nonlinear dynamical features of the discharge activity. The artificial extraction and the empirical judgment of the features were required for the recognition. Thus, these methods suffered from subjective factors and were not conducive to the identification of a large number of discharge rhythms.

**Results:**

The ability of automatic feature extraction along with the development of the neural network has been greatly improved. In this paper, an effective discharge rhythm classification model based on sparse auto-encoder was proposed. The sparse auto-encoder was used to construct the feature learning network. The simulated discharge data from the Chay model and its variants were taken as the input of the network, and the fused features, including the network learning features, covariance and approximate entropy of nerve discharge, were classified by Softmax. The results showed that the accuracy of the classification on the testing data was 87.5%, which could provide more accurate classification results. Compared with other methods for the identification of nerve discharge types, this method could extract the characteristics of nerve discharge rhythm automatically without artificial design, and show a higher accuracy.

**Conclusions:**

The sparse auto-encoder, even neural network has not been used to classify the basic nerve discharge from neither biological experiment data nor model simulation data. The automatic classification method of nerve discharge rhythm based on the sparse auto-encoder in this paper reduced the subjectivity and misjudgment of the artificial feature extraction, saved the time for the comparison with the traditional method, and improved the intelligence of the classification of discharge types. It could further help us to recognize and identify the nerve discharge activities in a new way.

**Supplementary Information:**

The online version contains supplementary material available at 10.1186/s12859-022-04592-3.

## Background

The neuron is the basic structure and functional unit of the nervous system. The nervous system can receive, transmit, and integrate information through abundant nerve discharge rhythms. Nerve discharge plays a key role in nerve information processing [1]. A typical neuron consists of a cell body (soma), dendrites, and an axon. There are ion channels constructed by pore-forming proteins embedded in the neuron membrane. These channels allow passage of ions through the channel pore and generate intracellular-versus-extracellular concentration differences of ions to establish the resting membrane potential. While the neuron's internal and external environment changes to a certain extent, the ion channels can generate an electrochemical pulse called an action potential (AP) by gating and controlling the flow of ions across the cell membrane. This movement of ions is an important part of maintaining life for an organism [2]. The action potentials and other electrical signals can travel rapidly along the neuron's axon, and activate synapses which are located at various points throughout the dendrites tree of the downstream neurons. Thus, the common nerve discharge phenomenon is generated [3].

Researches on the nerve discharge can be helpful to better understand the neuronal coding and neural information processing, and to reveal basics and underlying mechanisms of various neural activities. Different types of nerve discharges contain abundant and complex information [4]. Therefore, the identification of nerve discharges is the key to correctly understand the dynamical behavior of the nervous system. Nerve discharge was a complex non-linear phenomenon with many influencing factors and many nonlinear action modes, including such basic forms as random, chaotic, and periodic nerve discharge rhythms [5]. The development of nonlinear science, especially chaos theory, has provided abundant theoretical knowledge and analytical methods for the identification of nerve discharges. The relevant studies showed that the nonlinear time series analysis method could describe the randomness and certainty of the discharge rhythms and identify their signal sources [6]. However, over-reliance on the chaotic time series analysis methods can easily lead to misjudgments of the non-chaotic discharge rhythms, especially for the random rhythm. It was suggested that the characteristics other than nonlinear dynamic properties may also play a positive role in the identification of nerve discharge rhythms [7].

The simulation of the neuron model is also usually used to study the nerve discharge mechanism. Neuron models can make the complex influencing factors in the real biological neuron systems to be standardized, concrete and programmed. They are helpful to understand the mechanisms such as the generation of AP, the dynamics change of nerve discharges under different electric current stimulations, and so on [6]. Meanwhile, the simulation of the neuron model could effectively compensate for the shortcomings of complex and expensive animal experiments. Various nerve discharge rhythms were successfully simulated by using the same mathematical model under different parameter configurations [7, 8].

In both experimental and numerical simulation studies of neural activities, the following methods were often used to identify the nerve discharge rhythms: First, the observed discharge was fitted to the existing pattern by using the statistical descriptor or distribution of discharge activity. Second, based on the visualization of discharge sequence and the statistical histogram of interspike interval (ISI) [9], nonlinear time series analysis could characterize the randomness and certainty of discharge rhythms and identify their signal sources [10]. Therefore, according to several analysis indexes of ISI, certainty and randomness components were detected in neuronal discharge activities, which played a positive role to recognized and distinguished complex nerve discharge rhythms in the nervous system [11].

However, these methods with unknown parameters of nerve discharge mode were always subjective, as the parameters were usually estimated empirically by researchers or inferred from theoretical simulation data [5]. This made the feature extraction from the nerve discharge data with high nonlinearity and high dimensions more difficult [3]. And the excessive dependence on a few empirical indexes even caused confusion in the identification of nerve discharge mode [2]. Meanwhile, these methods were always time-consuming, as each discharge rhythm needed to be analyzed by various time series analysis methods artificially, and then each analysis result needed to be compared with the basic discharge types to determine the type of the testing discharge rhythm artificially [7]. In summary, it is difficult to accurately, objectively and fast add a class label to an unknown nerve discharge sequence. Therefore, it is essential to design a feature learning model that can automatically extract useful features from the long time nerve discharge series while keeping the feature compact.

Recently, deep learning rapidly developed and greatly improved the ability of the feature extraction methods in various fields, such as classification, recognition, and segmentation [12–17]. Such huge improvement is partially attributed to the feature extraction power of deep neural networks [18–21], especially in nonlinear feature extraction of high levels [22–25]. The sparse auto-encoder (SAE) is a special artificial deep neural network, which combines unsupervised learning and feature extraction [26–29]. It can effectively learn useful features from massive unlabeled data in an unsupervised way and show a powerful capability to extract nonlinear features from high-dimensional data. So it has been widely applied in many areas, like face recognition [30], complex industrial system monitoring [31], medical images recognition [32], radar image classification [33], wind speed forecasting [34] and so on. However, to the best of our knowledge, SAE, even deep neural networks, has not been applied in the basic nerve discharge classification tasks, neither in single neuron or neural fiber case.

The purpose of this study is to automatically extract some important features from the testing nerve discharge sequence and effectively classify the nerve discharge rhythms. Considering each nerve discharge sequence as a vector, an effective nerve discharge rhythm classification model based on SAE was proposed in this paper. The SAE was used to construct the feature learning network. The nerve discharge data, which was simulated by the commonly used theoretical neuronal firing model (Chay model) and its variants, were taken as the input of the network. The features learned were classified by the Softmax classifier. To combine supervised and unsupervised learning, two other common characteristics from testing time series were also fused with the features extracted from the SAE. Finally, by some restrictions on the hidden layer, the network could effectively learn the features that can best express the complex sample, the dimensions of the sample, and get a higher accuracy compared with other methods.

## Experiments and results

### Simulated dataset

The simulation data were obtained by the numerical simulation of the deterministic Chay model, stochastic Chay model with global Gauss white noise, stochastic Chay model with global Gauss color noise, stochastic Chay model with potassium ion Gauss white noise, stochastic Chay model with potassium ion Gauss color noise and improved deterministic Chay model. The simulation data set was composed of four types of nerve discharge: periodic discharge (PD) with category label 0, random discharge alternating with two periodic clusters discharge (RD) with category label 1, chaotic discharge (CD) with category label 2, and integer multiple discharge (IMD) with category label 3. Each discharge rhythm consisted of 60 ISI sequences with a length of 1024, of which 40 were training datasets (see Additional file 1) and 20 were testing datasets (see Additional file 2).

### Dataset theoretical model

Chay model was a multi-scale neuron system composed of fast and slow variables [2]. It was a typical ion channel model based on the Hodgkin-Huxley model to describe the complex nerve discharge rhythms. In previous studies, this neuronal firing model vividly restored real nerve discharge activities. As based on the ion channel dynamics, the key parameters in this model can correspond to the experimental operation well [4–9]. The model system was defined as:

1 $$\frac{dV}{{dt}} = g_{I} m_{\infty }^{3} h_{\infty } \left( {V_{I} - V} \right) + g_{K,V} n^{4} \left( {V_{K} - V} \right) + g_{K,C} \frac{C}{1 + C}\left( {V_{K} - V} \right) + g_{L} \left( {V_{L} - V} \right)$$

2 $$\frac{dn}{{dt}} = \frac{{n_{\infty } - n}}{{\tau_{n} }}$$

3 $$\frac{dC}{{dt}} = \rho \left( {m_{\infty }^{3} h_{\infty } \left( {V_{C} - V} \right) - K_{C} C} \right)$$

where, Eq. (1) represented the differential equation followed by the change rule of cell membrane potential, Eq. (2) represented the change rule of the probability of potassium channel opening depending on potential, and Eq. (3) represented the change rule of intracellular free calcium concentration.

When Gauss white noise $\xi \left( t \right)$ was add to Eq. (1), a stochastic Chay model with global Gauss white noise was constructed. When Gauss colored noise $\sigma \left( t \right)$ was added to Eq. (1), a stochastic Chay model with global colored noise was constructed. Other stochastic models were constructed in this way. The characteristics of Gauss white noise were as follows:

4 $$\left\langle {\xi \left( t \right)} \right\rangle = 0{ }$$

5 $$\left\langle {\xi \left( t \right),{\upxi }\left( {t^{\prime } } \right)} \right\rangle = 2D\delta \left( {t - t^{\prime } } \right)$$

*D* was noise intensity and $\delta$ was the Dirac-delta function.

The characteristics of Gauss colored noise were as follows:

6 $$\frac{d\sigma }{{dt}} = - \frac{1}{\tau }\sigma + \frac{{\sqrt {2D} }}{\tau }\xi \left( t \right)$$

7 $$\left\langle {\sigma \left( t \right)} \right\rangle = 0$$

8 $$\left\langle {\sigma \left( t \right),{\upsigma }\left( {t^{\prime } } \right)} \right\rangle = D/\tau *e^{{ - \frac{{t - t^{\prime } }}{\tau }}}$$

The Gauss white noise of potassium ion and the Gauss color noise of potassium ion were added to Eq. (2) to form a stochastic Chay model. The Gauss white noise of potassium ion was described as follows:

9 $$\eta_{n} = \xi n\left( t \right)\frac{1}{{\sqrt {NK} }}\sqrt {\frac{2}{\tau n\left( V \right)}n\left( {1 - n} \right)}$$

where $\xi_{n} \left( t \right)$ was Gauss white noise, *N* was the number of ion channels, $\tau_{n} \left( V \right)$ was the relaxation time of ion channels, and *n* was the probability of opening ion channels. When $\xi_{n} \left( t \right)$ was Gauss color noise, $\eta_{n}$ was Gauss color noise of potassium ion.

In our previous studies, a biological fact was considered that when the action potential voltage reaches its peak, the *K*^+^ channel opened completely instantaneously at the point between the end of depolarization and the beginning of repolarization. On this basis, the improved deterministic Chay model was proposed. The biological process was expressed as:

10 $$\frac{dn}{{dt}} = w_{K} \frac{{n_{\infty } - n}}{{\tau_{n} }}$$

The parameter $w_{K}$ controlled the process. Equations (1), (3) and (10) constituted an improved deterministic Chay model. For more information, please refer to Ref. [10].The improved model could simulate the nerve discharge type with random rhythm without noise, which rhythm could not be simulated in the original deterministic Chay model and only observed in the stochastic Chay model before.

Using the above models, the discharge sequences could be simulated, and the ISI time series could be transformed from the spike trains.

## Parameter analysis and valuation indicators

### Network parameters

The structure and training parameters of the SAE in this paper were shown in Table 1.

**Table 1:** Network parameter settings of the SAE

Parameter name	Parameter value
Number of input layer nodes	1024
Number of output layer nodes	4
Number of nodes in layer 1	1224
Number of nodes in layer 2	824
Unsupervised training epochs	1000
Supervised training epochs	1000
*L*_2_ weight regularization	0.01
Loss function	Crossentropy
Sparsity regularization *β*	0.1
Training algorithm	Trainscg

### Confusion matrix (CM)

CM, also known as error matrix, was a standard form of precision evaluation, which expressed in *N* rows and *N* columns [35]. Each column of the CM represented the actual category, and each row represented the prediction category. The value of each column represented the number of data predicted for this class.

Through the CM, the following equation could be obtained:

11 $${\text{Overall }}\;{\text{Accuracy}} = \frac{{\mathop \sum \nolimits_{i = 1}^{k} N_{ii} }}{{\mathop \sum \nolimits_{i = 1}^{k} \mathop \sum \nolimits_{j = 1}^{k} N_{ij} }}$$

The overall accuracy was the proportion of all correctly judged results in the total observation value of the classification model. *N*_*i j*_ represented the number of discharge sequences of class *i* predicted to class *j*. *N*_*i,i*_ represented the number of discharge sequences of class *i* predicted to class *i*.

## Results

We validated the SAE in this paper on the simulation datasets, which contained four basic types of nerve discharge. The total training time was 17 min and the test time was 15 s. With the same data size, the time consumption of traditional artificial classification method was at least 30 min. The CM on the testing datasets classified by only the automatically extracted features from stacked SAE was shown in Table 2.

**Table 2:** CM on the testing datasets classified by the features from stacked SAE

Type	PD_p_	RD_p_	CD_p_	IMD_p_	Overall accuracy (%)
PD_t_	15	3	2	0	68.75
RD_t_	4	2	11	3	
CD_t_	2	0	18	0	
IMD_t_	0	0	0	20	

The subscript p and t represent the predicted value and the true value respectively

The results showed that the overall accuracy was ACC = 68.75%. All the IMD rhythms were predicted correctly, and almost all the RD rhythms were classified incorrectly. Most of them were classified into CD rhythms. Therefore, we considered adding some important statistical features to the network manually and then observed the classification results. As a classical statistical index to measure the overall error of variables, covariance was widely used in neuroscience [36–39]. In this paper, we merged the covariance (the calculation method was described in Ref. [38]) of discharge data with the features extracted automatically by the stacked SAE and trained them in the Softmax classifier. The testing data were validated and the CM was obtained as shown in Table 3.

**Table 3:** CM on the testing datasets classified by the features from stacked SAE and *covariance*

Type	PD_p_	RD_p_	CD_p_	IMD_p_	Overall accuracy (%)
PD_t_	15	3	2	0	76.25
RD_t_	5	8	7	0	
CD_t_	2	0	18	0	
IMD_t_	0	0	0	20	

The subscript p and t represent the predicted value and the true value respectively

The results showed that after incorporating the covariance of discharge data, the overall accuracy was increased from 68.75 to 76.25%. The accuracy of classification of RD rhythms was improved, while the classification results of other rhythms remained unchanged. However, the improvement to the classification result of RD rhythms with covariance fused was limited. As a complexity measure suitable for short data, approximate entropy was frequently applied to feature description of neural discharge data [4–7]. So the approximate entropy (ApEn, the calculation method was described in Ref. [7]) of discharge data was also merged with the features extracted automatically by the stacked SAE and then trained them in the Softmax classifier. The testing data were validated and the CM was obtained as shown in Table 4.

**Table 4:** CM on the testing datasets classified by the features from stacked SAE and *ApEn*

Type	PD_p_	RD_p_	CD_p_	IMD_p_	Overall accuracy (%)
PD_t_	16	2	2	0	72.5
RD_t_	5	5	10	0	
CD_t_	2	1	17	0	
IMD_t_	0	0	0	20	

The subscript p and t represent the predicted value and the true value respectively

The results showed that after incorporating the ApEn of the discharge data, the overall accuracy was increased from 68.75 to 72.5%. The introduction of ApEn to the features learned from the stacked SAE further improved the classification of RD rhythms. But simultaneously, the number of CD sequences correctly classified declined. Therefore, we merged the ApEn and covariance of discharge data with the features automatically extracted by the stacked SAE and trained them in the Softmax classifier. The testing data were validated, and the CM was showed in Table 5.

**Table 5:** CM on the testing datasets classified by the features from stacked SAE, *covariance* and *ApEn*

Type	PD_p_	RD_p_	CD_p_	IMD_p_	Overall accuracy (%)
PD_t_	17	1	2	0	87.5
RD_t_	2	17	1	0	
CD_t_	1	3	16	0	
IMD_t_	0	0	0	20	

The subscript p and t represent the predicted value and the true value respectively

The results showed that the overall accuracy was significantly increased with a value of 87.5%. Especially, by the fine-tuning with these fused features, the Softmax classifier obtained better training and correctly identified more RD sequences. Thus the results suggested that the fusion of the covariance, ApEn with the features extracted automatically by the stacked SAE, could effectively distinguished and identified the different types of nerve discharge rhythms, which was based on the good classification effect.

Table 6 summarized the classification results of different methods on the dataset. Compared with other methods, the accuracy of this novel method is higher. A model was proposed and trained in this work to learn the automatic feature extraction from the discharge sequence. The model effectively reduced the subjectivity, the time consumption and other adverse factors of the previous methods. At the same time, cov and ApEn of the discharge sequence were integrated as a new feature, which was introduced into the model, and then classified. The comparison results showed that these two characteristics of the discharge sequence had a great influence on the classification results, especially on the results of RD discharge rhythm.

**Table 6:** Performances of different methods to classify nerve discharge rhythms

Method	Overall accuracy (%)
KNN	65.00
SVM	68.75
SAE	68.75
Our proposed method	87.50

## Discussion

The nerve discharge rhythms, which constructed the classification experiment dataset of the present study, were simulated by the original deterministic Chay model and its variants, including stochastic Chay model with global Gauss white noise, stochastic Chay model with global Gauss color noise, stochastic Chay model with potassium ion Gauss white noise, stochastic Chay model with potassium ion Gauss color noise and improved deterministic Chay model. The improved deterministic Chay model based on the rational biological fact had a strong ability to simulate abundant nerve discharge forms. It could simulate not only the rhythms simulated by the original Chay model, but also the rhythms simulated by the stochastic Chay model with noise. Various complex discharge rhythms would be observed in a same dynamical bifurcation process. This improved model may provide the possibility to understand the unity of the uncertainty and randomness of the neuron system in a new light, and further enrich the understanding of the mechanism of neuron multi-peak discharge modes.

In this work, a learning neural network model based on SAE was proposed for the automatic classification of nerve discharge rhythms. In fact, the auto-encoder (AE) itself has powerful abilities of unsupervised feature extraction and has a great advantage in vector processing. The more neurons exit in the hidden layer, the stronger expression ability the model has and the more complex features could be learned. But too many hidden neurons would easily lead to over-fitting, effecting the generalization ability of the network. This was the reason why we chose the stacked sparse auto-encoder. Without sparsity, the input couldn't be correctly expressed in compression. Specifically, if sparse constraints were added to hidden neurons, the auto-encoder neural network could find some interesting structures in the input data even in the case of a large number of hidden neurons, and effectively avoided the over-fitting. The results showed that the feature of the nerve discharge rhythms could be really automatically extracted by the SAE learning, especially accurately for the IMD and PD rhythms. This model exactly classified the nerve discharge rhythms more intelligently with low time-consuming than the previous methods.

However, the accuracy obtained by only the SAE model was low as the RD rhythms were not well distinguished from the CD rhythms. In fact, these two discharge rhythms were quite confounding in both previous biological experiment and numerical simulation studies, in which the statistical characteristics and the nonlinear characteristics of these two rhythms are very similar. Considering the combination of supervised and unsupervised classification, a statistical characteristic "covariance" and a nonlinear characteristic "ApEn" often used in previous analysis were fused additively with the features extracted by the second SAE. The covariance was a measurement of dispersion, and revealed the degree of fluctuation among samples. The ApEn could describe the complexity of the discharge sequences, and detect determinate components and random components in the mixed information. The larger value of ApEn indicated the higher complexity and the stronger randomness of the discharge sequence. It was found that the accuracy was significantly improved after incorporating these two features, indicating that the model was helpful for the classification of nerve discharge and improved the reliable quantitative evaluation for the recognition of nerve discharge rhythms.

These results also suggested that the classification model based on SAE proposed in this paper still had limitations to identify the nerve discharge rhythms with more disorder, although its advantages were significant. Because of the high complexity, irregularity, nonlinearity and difference of the nerve discharge activities, it was difficult to classify the nerve discharge rhythms by a single method or simple methods. These were also the reason that the time-consuming, empirical and subjective combined methods were used in the past but couldn't get a satisfying result. The final results of this work also provided a new effective approach to classify the more complex or unknown nerve discharge rhythms, that was integrating some simple features to the classification model based on SAE.

## Conclusions

In this study, an automatic classification of nerve discharge rhythms based on sparse auto-encoder and time series feature was proposed. A stacked sparse auto-encoder was constructed by integrating two SAEs to realize the automatic extraction of nerve discharge features. A Softmax classifier was integrated on the top of the stacked sparse auto-encoder to complete the automatic classification of simulated nerve discharge rhythms. The time series feature of the simulated nerve discharge sequence was fused with the feature extracted from the SAE, and significantly improved the accuracy of classification results. In addition, the improved deterministic Chay model based on the generation mechanism of AP was used to simulate the nerve discharge in this paper. This model showed great ability for simulation and promoted the construction of simulation datasets.

SAE was firstly used to classify the basic types of discharge data from a neuron or a single nerve fiber, and achieved automatically extract the characteristics of the nerve discharge. This model reduced the time of artificial judgment, avoided the empiricism of traditional methods, and made the classification of nerve discharge types more intelligent. This work could provide new viewpoints and relatively reliable methods for the recognition of nerve discharge.

The final goal of this method proposed is to solve the identification of real biological nerve discharge rhythms. However, the real data from neurophysiological experiment is very difficult to obtain, as experimental instrument, experimental operator, and even experimental reagent may become restrictive factors. With the accumulation of real data, the method proposed in this paper will be validated and improved in the further studies.

## Methods

### Preprocessing

As the parameters of neuron models, which were set by researchers, were different during simulation, the length of the nerve discharge sequence and the size of the ISI obtained would also be different. Therefore, we normalized the discharge data and controlled the length of the discharge sequence at 1024. This length was also frequently used to analyze the nerve discharged data in many experimental and clinical studies.

### Sparse auto-encoder (SAE)

**Fig. 1:**
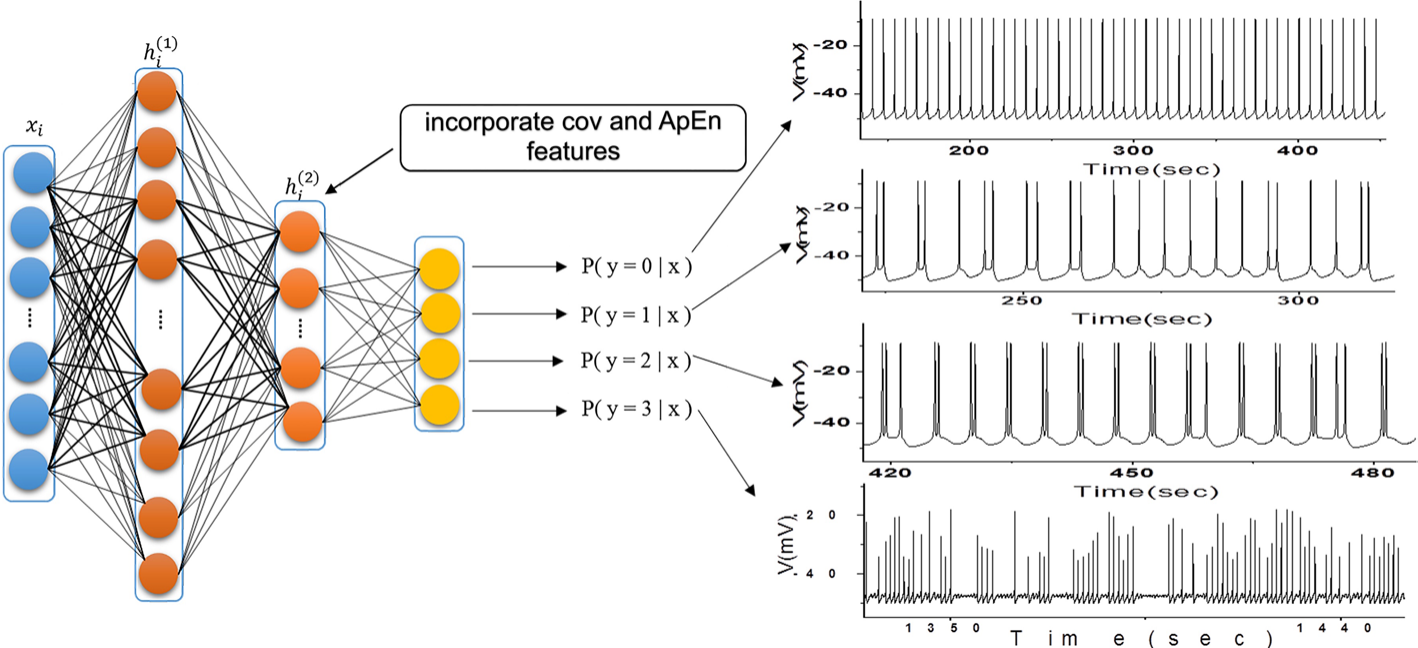
Neural network architecture of the stacked SAE and nerve discharges corresponding to the classification results. The left part of the figure shows the structure of the stacked SAE. The right part of the figure shows the different nerve discharges corresponding to the classification results. The text illustrates the added statistical features to tune the training process

The stacked sparse automatic encoder used in this paper was made up of two SAEs superimposed [32]. Its network structure consisted of four layers, including an input layer, two hidden layers, and an output layer, as shown in Fig. 1. The 1024-dimensional axial data was input into the model as input data to obtain depth representation, and then the classifier was trained according to the representation and its corresponding labels.

As an unsupervised nonlinear neural network, the SAE was trying to make the output of the model infinitely close to the model input, so that the feature representation of the model was more robust. The encoding process was from the input layer to the hidden layer, and the decoding process was from the hidden layer to the output layer. In encoding, the input vector *x* was mapped by function $h = f^{\left( 1 \right)} \left( {W^{\left( 1 \right)} x + b^{\left( 1 \right)} } \right)$, and the hidden layer representation was obtained. In decoding, the representation *h* of hidden layer was re-mapped by function $\hat{x} = f^{\left( 2 \right)} \left( {W^{\left( 2 \right)} x + b^{\left( 2 \right)} } \right)$ to obtained reconstructed data. The model was optimized by minimizing the cost function of reconstruction error, and the cost function was defined as the mean square error function:

12 $$J\left( {W,b} \right) = \left[ {\frac{1}{m}\mathop \sum \limits_{i = 1}^{m} \left( {\frac{1}{2}f\left( {x^{\left( i \right)} - \hat{x}^{\left( i \right)} } \right)_{2}^{2} } \right)} \right] + \lambda \times \Omega_{weights} + \beta \times \Omega_{sparsity}$$

${\Omega }_{weights}$ was the *L*_*2*_ regular constraint, ${\Omega }_{sparsity}$ was the sparse restriction.

For each SAE, Eq. (12) was used to minimize the difference of features to optimize network parameters and obtained representative depth features. In the training process, the gradient descent method was used to optimize the loss function.

Then, in the stacked sparse auto-encoder constructed by two SAE models, the output of the SAE on the previous level served as the input of the SAE on the next layer. More and deeper abstract information from data could be obtained. However, the deep information learned from stacked sparse auto-encoder was adaptive and could not classify. Therefore, to effectively classify the learned features, a classifier was integrated at the top of the stacked sparse auto-encoder to complete the training of the classification model. Sometimes, several statistical features would be fused with the learned features by stacked SAE to fine-tune the training of classifier.

### Softmax classifier

When SAE was used in the classification field, a robust classifier was often added to the last layer of the network. Since this work was a multi-category classification experiment, Softmax classifier was used for classifying to make the network application more extensive and reach more accurate results [34].

For training sets {{*x*^(1)^, *y*^(1)^}, {*x*^(2)^, *y*^(2)^},…, {*x*^(m)^, *y*^(m)^}}, label *y* was assigned *k* different values to represent *k* categories, and *p*( *y* = *j* | *x*) was assumed to represent the probability that the sample is determined as category *j* when input *x*. Therefore, for a *k*-class classifier, the classification result was a *k*-dimensional vector, and the classification result was expressed as:

13 $$h_{\theta } \left( {x^{\left( i \right)} } \right) = \left[ {\begin{array}{*{20}c} {p\left( {y^{\left( i \right)} = 1 | x^{\left( i \right)} ;\theta } \right)} \\ {p\left( {y^{\left( i \right)} = 2 | x^{\left( i \right)} ;\theta } \right)} \\ \cdots \\ {p\left( {y^{\left( i \right)} = k | x^{\left( i \right)} ;\theta } \right)} \\ \end{array} } \right] = \frac{1}{{\mathop \sum \nolimits_{j = 1}^{k} \exp \left( {\theta_{j}^{T} x^{\left( i \right)} } \right)}}\left[ {\begin{array}{*{20}c} {\exp \left( {\theta_{1}^{T} x^{\left( i \right)} } \right)} \\ {\exp \begin{array}{*{20}c} {\left( {\theta_{2}^{T} x^{\left( i \right)} } \right)} \\ \cdots \\ \end{array} } \\ {\exp \left( {\theta_{k}^{T} x^{\left( i \right)} } \right)} \\ \end{array} } \right]$$

where, *θ* was a matrix of k rows, and each row corresponded to the parameters of the classifier. $\frac{1}{{\mathop \sum \nolimits_{j = 1}^{k} {\text{exp}}\left( {\theta_{j}^{T} x^{\left( i \right)} } \right)}}$ was the normalization of probability distribution so that the sum of all probabilities was 1. Therefore, the cost function of the Softmax classifier was defined as:

14 $$J\left( \theta \right) = - \frac{1}{m}\mathop \sum \limits_{i = 1}^{m} \mathop \sum \limits_{j = 1}^{k} 1\left\{ {y^{\left( i \right)} = j} \right\}log\frac{{\exp \left( {\theta_{j}^{T} x^{\left( i \right)} } \right)}}{{\mathop \sum \nolimits_{l = 1}^{k} \exp \left( {\theta_{l}^{T} x^{\left( i \right)} } \right)}}$$

In the above formula: 1{·} was the indicator function, that was, 1{true} = 1, 1{false} = 0.

## Supplementary Information

**Additional file 1**.** Training datasets**: Training datasets composed of different types of ISI sequences simulated by theoretical models, and the Category labels of Training datasets.

**Additional file 2**.** Testing datasets**: Testing datasets composed of different types of ISI sequences simulated by theoretical models, and the Category labels of Training datasets.

## Data Availability

The datasets used during the present study are available from Additional Files.

## Abbreviations

AE: Auto-encoder
AP: Action potential
ApEn: Approximate entropy
CD: Chaotic discharge
CM: Confusion matrix
IMD: Integer multiple discharge
ISI: Interspike interval
PD: Periodic discharge
RD: Random discharge alternating with two periodic clusters discharge
SAE: Sparse auto-encoder
